# A General View of the Writings of Linnæus

**Published:** 1782

**Authors:** 


IV. *A General View of the Writings of Linnæus.* *By* Richard Pulteney, M. D. and F.R.S. 8vo, Payne, *London.* 1781. 425 pages. 6s. boards.

WE have here a concife and judicious view of the writings of a Profeffor, whofe talents enabled him to reform the whole fcience of natural hiftory.

The work begins with fome biographical anecdotes, which, as the author modeftly informs us, have been almoft wholly collected from Linnæus's own writings, and other printed works, and are introduced principally to relieve the tedioufnefs of a bare account of books, and to connect in a better manner the feries and occafions of his publications, all of which are noticed in this performance; but as moft of them were fubfervient to his great object the *Syftem of Nature*, the outlines of that work bear a principal part in the volume before us.

Charles von Linné, we are told, was the fon of a Swedifh divine, and born May 24, 1707, at Roefhult in the province of Smaland, in Sweden. His father was foon after preferred to the living of Stenbrihuet, in the fame province, where where dying in 1748, at the age of 70, he was ſucceeded by another ſon. The family took their name from a large lime or linden tree, yet ſtanding on the farm where Linnæus was born.

From the ſtraightneſs of his father's income, our young naturaliſt was on the point of being deſtined to a mechanical employment; but fortunately this deſign was over-ruled. In June, 1717, he was ſent to ſchool at Wexſio, from whence he was removed to Lund, and from this laſt place in 1728 to Upſal, where he ſoon contracted a cloſe friendſhip with Artedi, one of his fellow-ſtudents, whoſe name is ſo famous in Ichthyology.

Olaus Celſius, who was the reſtorer of natural hiſtory in Sweden, being ſtruck with the diligence of Linnæus, admitted him to his houſe, his table, and his library. In 1708 he was deputed by the Academy of Sciences at Upſal to make the tour of Lapland. This tour had been made for the firſt time by the elder Rudbeck in 1695, but unfortunately the whole fruit of that expedition, except two or three copies of the *Campi Elyſii*, periſhed in the dreadful fire of Upſal, in 1702. In this journey, the greateſt part of which was performed on foot, Linnæus though though young and robuſt, was frequently quite exhauſted. He was wont to ſleep under the boat with which he and his companions (two Laplanders, one his interpreter, the other his guide) forded the rivers. In deſcending one of theſe rivers, he narrowly eſcaped periſhing by the overſetting of the boat, and loſt many of the natural productions which he had collected.

After his retturn to Upſal, he preſented a *florula Lapponica* to the Academy, in which he arranged the plants according to the ſexual ſyſtem. In 1733 he viſited the ſeveral mines in Sweden, and the year following was employed to inveſtigate the natural productions of Dalekarlia, where he taught mineralogy and practiſed phyſic. Here he contracted an acquaintance with the daughter of a Dr. More, phyſician of the place, whom he afterwards married.

In 1735 Linnæus travelled over many other parts of Sweden, ſome parts of Denmark and Germany, and fixed in Holland, where* he took his doctor's degree in phyſic in the month of June of that year. His theſis on that occaſion was entitled *Hypotheſis nova de febrium intermittentium*

* At Harderwyck.

*mittentium causa.* In this year also he published his first sketch of the *Systema Naturæ*, in 12 pages in folio. The year following he came to England, where he was well received by Dillenius, Dr. Martyn, Mr. Rand, Mr. Millar, Mr. Collinson, and particularly by Dr. Isaac Lawson. Boerhaave's letter to Sir Hans Sloane on this occasion is preserved in the British Museum, and runs thus: "*Linnæus qui has tibi dabit li-" "teras, est unice dignus te videre, unice dignus a" "te videri; qui vos videbit simul, videbit homi-" "num par, cui simile vix dabit nobis.*" The coolness with which he was received by Sir Hans, was probably (as our author suspects) owing to the attachment of the latter to Ray's method. Linnæus speaking afterwards of London, in a letter to a friend, called it, "*Punctum" "saliens in vitello orbis.*"

To persons who with great merit possess but little advantages of fortune, it may not be unpleasant or unprofitable, to observe the slender beginning from which Linnæus rose to ease and affluence in life. "*Exivi patria triginti sex" "nummis aureis dives,*" are his own words. Boerhaave, who knew how to appreciate his talents, recommended him to the patronage of Mr.

Mr. Clifford, with whom he reſided for a conſiderable time near Harlem. The ſame celebrated Profeſſor afterwards recommended him to be phyſician at Surinam, but Linnæus declined accepting the offer, on account of his having been educated in ſo oppoſite a climate. At his requeſt, however, the appointment was given to a young German phyſician of great merit, who had the misfortune to fall a ſacrifice partly to the climate, and partly perhaps to ill uſage from the governor, in half a year after his arrival: a circumſtance which Linnæus has very pathetically lamented in the *Flora Suecica*, when treating of a plant to which he has given this gentleman's name.

Early in 1738 he viſited Paris, but previous to this journey he had publiſhed not only his *Syſtema Naturæ*, but likewiſe 1. his *Fundamenta Botanica*, in which the ſcience of Botany is reduced to 365 aphoriſms. It paſſed through ſeveral editions, and was publiſhed with a comment on each aphoriſm in 1751, under the title of *Philoſophia Botanica*. 2. *Bibliotheca Botanica*, in which botanic writers are diſtributed into ſixteen claſſes. 3. *Genera Plantarum*, which our author conſiders as one of the moſt capital of

Linnæus's works. We are told that before the publication of the firſt edition he had examined the characters of 8000 flowers. "Thoſe alone, obſerves Dr. Pultney, who have been accuſtomed to examine plants with a ſcientific view, can judge how arduous this undertaking muſt have been." The firſt edition of this book contained 935 genera; the ſixth and laſt in 1764, 1249; and the Mantiſſæ have ſince extended the number to 1336. The flowering of the Plantain or Banana, in Mr. Clifford's garden, induced him to give a complete deſcription of that plant, which is a model for monographers in this way. The ſame year likewiſe (1737) produced the *Corollarium Generum cui accedit Methodus Sexualis*—the *Flora Lapponica*—and the *Critica Botanica.* Ludwig, when ſpeaking of the latter of theſe ſays "*rigoroſus quidem, ſed* "*ſæpiſſimè felix botanicorum cenſor eſt.*"

In 1737 alſo he publiſhed, at the expence of his patron, the *Hortus Cliffortianus*, the moſt ſplendid of all his writings. The drawings for it were made by Ehret. Of this work Dr. Pultney obſerves, that from the copious number of ſynonyms, it is almoſt a pinax of every plant therein contained.

The

The laſt performance of his own which Linnæus publiſhed in Holland, was his *Claſſes Plantarum*, which is a very large illuſtration of the ſecond part of his *Fundamenta*, and contains a compendious view of all the ſyſtems of botany.

Linnæus, whilſt in Holland, loſt his friend Artedi, whoſe papers he with ſome difficulty procured and publiſhed in 1738, in 8vo. Artedi was unfortunately drowned in one of the canals of Amſterdam on his return from England, where he had been to perfect his ſyſtem.

Toward the latter end of 1738, Linnæus returned to Sweden, and ſettled as a phyſician at Stockholm, where he met with conſiderable oppoſition, and was oppreſſed with many difficulties, all of which at length he overcame, and got into extenſive practice. He was appointed phyſician to the Admiralty, and had a ſtipend from the citizens for giving lectures in botany. The Royal Academy of Sciences inſtituted in 1739, choſe him their firſt preſident, and in 1741, upon the reſignation of Roberg, he was appointed joint profeſſor of phyſic and phyſician to the King with Roſen, who had been nominated the preceding year on the death of Rudbeck. Roſen undertook to teach anatomy, phyſiology, pathology, and therapeutics; Linnæus, natural hiſtory, botany, materia medica, the dietetic part, and the diagnoſtics.

During the interval of his removal from Stockholm to Upſal, he was deputed by the ſtates of the kingdom to make a tour to the iſlands of Oeland and Gothland, in order to make ſuch inquiries as might tend to improve agriculture and the arts. On his return, he entered upon his profeſſorſhip, and on this occaſion pronounced his oration '*de Peregrinationum intra patriam neceſſitatem*,' Oct. 17, 1741. In 1745, his *Iter Oelandicum et Gothlandium* was printed at Stockholm, as was alſo his *Iter Scanicum.* As a proof of the little attention that had been paid to natural hiſtory in Sweden, it is obſerved that in the former of theſe journies he diſcovered above 100 plants, which before were not known to be indigenous; many of them ſuch as are uſed in phyſic and in dyeing. In the *Iter Oelandicum* there occurs a curious remark on vegetation, confirming the annual increaſe of wood in an oak tree, in which were perfectly diſtinguiſhed the hard winters of 1568, 1787, and 1709, by the narrow circles in thoſe years.

Soon

Soon after his eſtabliſhment at Upſal he laboured to get the botanic garden put on a better footing. At that time it did not contain above fifty exotics, but his correſpondence with the firſt botaniſts in Europe ſoon ſupplied him with great variety. He received Indian plants from Juſſieu and Van Royen; European plants from Haller and Ludwig; American ones from Collinſon and Cateſby: and a variety of annuals from Dillenius, ſo that from his *Hortus Upſalienſis* publiſhed in 1748, it appeared that he had introduced 1100 ſpecies, excluſive of Swediſh plants and varieties.

In 1745 appeared his *Flora Suecica*, and in 1746 his *Fauna Suecica*. About this period he received an *Herbarium* collected by the famous Dr. Paul Herman. This collection had been loſt for upwards of fifty years, until chance threw it into the hands of Mr. Gunther, apothecary to the King of Denmark, who ſent it to Linnæus, with a requeſt that he would examine it, which he did, and was thereby enabled to form many new genera, and to ſettl many doubtful ſpecies. He publiſhed the reſult of his laborious reſearches under the title of *Flora Zeylanica*, illuſtrated with figures of upwards of 200 of theſe

 plants.

plants. Linnæus authenticates this herbarium to have been Herman's, by ſhewing that the numbers and the plants anſwer to his *Muſæum Zeylanicum*, publiſhed in 1717.

In 1749 Linnæus publiſhed his *Materia Medica* for the uſe of his ſtudents. The ſame year too appeared the firſt volume of Theſes in 8vo. under the title of *Amœnitates Academicæ*, of which ſix other volumes have ſince been publiſhed. As theſe academical diſſertations were ſuſtained by Linnæus in his profeſſorial character, and were ſelected by himſelf, they have been regarded as of equal authority with his own writings.

In 1751 while Linnæus was meditating one of his principal works, he had a long and painful fit of the gout. Upon the recovery of his health, he publiſhed his *Philoſophia Botanica*, which may be conſidered as the inſtitutions of the Linnæan ſyſtem of botany. In this work, ſays Dr. Pulteney, it is difficult to determine whether we ought moſt to admire the genius of the author in his inventive power, or that exquiſite arrangement which he has given to the whole.

In 1753 appeared the *Species Plantarum*, which Dr. Pulteney calls '*opus maximum et æternum*'.

'num.' To give this work its utmoſt perfection had been the author's object for many years, and to this all his other productions are in ſome meaſure only preparatory. As this work contains all the plants of the known world which had come to Linnæus's knowledge, or rather inſpection (as he ſeldom admits any on the authority of others) and which at the publication of theſe volumes appear to have amounted to 7,300 ſpecies, all varieties excluded, Dr. Pulteney regrets that it could not have been extended by the author himſelf to a complete pinax and hiſtory of every plant therein deſcribed.

In this year alſo Linnæus publiſhed his *Muſeum Teſſinianum*, or a deſcription of the cabinet of his firſt patron and great friend Count Teſſin. In 1754 he did the ſame office for the Royal Muſeum.

We now begin to ſee him in a more elevated ſituation in life. His reputation had already procured him honours from almoſt all the Royal Societies in Europe, and his own ſovereign had created him a Knight of the Polar Star. It was now no longer *laudatur et alget.* His practice in his profeſſion became lucrative, and we find him ſoon after poſſeſſed of his country houſe and

 gardens gardens at Hammarby, five miles from Upfal. He had moreover received a flattering teftimony of the extent of his fame. This was an invitation to Madrid from the King of Spain, there to prefide as a naturalift, with the offer of an annual penfion for life of 2000 piftoles, letters of nobility, and the free exercife of his own religion. To this fingular honour, after the moft perfect acknowledgements, he returned for anfwer, "that if he had any merits, they were due to his own country."

In 1755 the Royal Academy at Stockholm honoured him with one of their firft premiums. In 1759 he alfo obtained the prize of 100 ducats from the Academy at St. Peterfburgh, for the beft paper written to eftablifh or difprove, by new arguments, the doctrine of the fexes of plants. This was the more honourable to him, as a Profeffor of that Academy had a few years before endeavoured to overturn the Linnæan fyftem.

The great character of Linnæus and his colleagues, particularly of Rofen, raifed the credit of the univerfity of Upfal fo much, that the number of ftudents was nearly double what it is faid to have been thirty or forty years before.—

The

The travels of many of his pupils afforded him great sources of information, and enabled him to enlarge and improve his System of Nature, which in the 12th and last edition is enlarged from 226 to 1327 pages.

After giving a very full account of this publication, Dr. Pulteney proceeds to consider his *Genera Morborum*. This classification of diseases constitutes but a small part of his works, yet as Linnæus was an early writer on this subject, which has since excited the attention of many physicians, and is at this day not sufficiently discussed, Dr. Pulteney has thought proper to exhibit it more largely than many of his other writings. In this performance Linnæus has nearly retained the arrangement of Sauvages, although he has altered his terms and constituted one more class, the exanthematic or eruptive fever, which in the systems of Sauvages and Cullen, form an order or subdivision of a class.

In 1776 he published a small piece entitled *Clavis Medicinæ duplex, exterior et interior*, which may be considered as a syllabus of his lectures. In 1771 appeared his last work under the title of *Mantissa altera*.

In the preface to his *Flora Lapponica* he had given hopes of a compleat natural hiſtory of that country, under the title of *Lacheſis Lapponica.* Mr. Pennant once reminded him of it, and received for anſwer:

> Nunc nimis ſero inciperem:

> Me quoque debilitat ſeries immenſa laborum,

> Ante meum tempus cogor et eſſe ſenex

> Firmâ ſit illa licet; ſolvetur in æquore navis,

> Quæ nunquam liquidis ſicca carebit aquis.

It appears that Linnæus enjoyed upon the whole a good conſtitution. At times, however, he was ſubject to hemicrania and gout. In 1776, his infirmities increaſed apace. At the latter end of that year he was ſeized with an apoplexy, which left him paralytic; and at the beginning of 1777 he ſuffered another ſtroke, which very much impaired his mental powers. But the diſeaſe, which was the more immediate cauſe of his death, was an ulceration in his urinary bladder. He died January 11, 1778, aged 70 years and 8 months, leaving a widow, a ſon, and five daughters.

Elizabeth Chriſtina, one of his daughters, made herſelf known to the learned world in 1762, by by a diſcovery which was publiſhed in the Swediſh Acts of the ſame year. It related to a curious, and before quite unobſerved appearance in the flowers of the Indian creſſes, which ſhe had perceived to emit ſpontaneouſly, at certain intervals, ſparks like thoſe of electricity. This was only viſible in the duſk of the evening, and ceaſed when total darkneſs came on. Mr. Wilcke conſidered it as an electrical phænomenon.

The memoirs of Linnæus and his writings are followed by a brief account of the contents of the *Amænitates Academicæ*, and a tranſlation of the *Pan Suecus*. This laſt was preſented to the Engliſh reader by our author ſeveral years ago in the Gentleman's Magazine, but is here reprinted with additional obſervations, and ſome improvements in the general arrangement of the tables.

The work cloſes with a catalogue of the writings of Linnæus.

