# Errata

**Published:** 1782

**Authors:** 


					E R R A T A.
Pa^r 31, line. 9 from the top, f r rettum rea.-l return ;

				

